# Adult Raphe-Specific Deletion of *Lmx1b* Leads to Central Serotonin Deficiency

**DOI:** 10.1371/journal.pone.0015998

**Published:** 2011-01-05

**Authors:** Ning-Ning Song, Jian-Bo Xiu, Ying Huang, Jia-Yin Chen, Lei Zhang, Lise Gutknecht, Klaus Peter Lesch, He Li, Yu-Qiang Ding

**Affiliations:** 1 Department of Anatomy and Neurobiology, Tongji University School of Medicine, Shanghai, China; 2 Molecular and Clinical Psychobiology, Department of Psychiatry and Psychotherapy, University of Würzburg, Würzburg, Germany; 3 Key Laboratory of Nervous System Diseases, Ministry of Education of China, Division of Histology and Embryology, Department of Anatomy, Tongji Medical College, Huazhong University of Science and Technology, Wuhan, China; Université de Toulouse, France

## Abstract

The transcription factor *Lmx1b* is essential for the differentiation and survival of central serotonergic (5-HTergic) neurons during embryonic development. However, the role of *Lmx1b* in adult 5-HTergic neurons is unknown. We used an inducible Cre-LoxP system to selectively inactivate *Lmx1b* expression in the raphe nuclei of adult mice. *Pet1-CreER^T2^* mice were generated and crossed with *Lmx1b^flox/flox^* mice to obtain *Pet1-CreER^T2^*; *Lmx1b^flox/flox^* mice (which termed as *Lmx1b* iCKO). After administration of tamoxifen, the level of 5-HT in the brain of *Lmx1b* iCKO mice was reduced to 60% of that in control mice, and the expression of tryptophan hydroxylase 2 (*Tph2*), serotonin transporter (*Sert*) and vesicular monoamine transporter 2 (*Vmat2*) was greatly down-regulated. On the other hand, the expression of dopamine and norepinephrine as well as aromatic L-amino acid decarboxylase (*Aadc*) and *Pet1* was unchanged. Our results reveal that *Lmx1b* is required for the biosynthesis of 5-HT in adult mouse brain, and it may be involved in maintaining normal functions of central 5-HTergic neurons by regulating the expression of *Tph2*, *Sert* and *Vmat2*.

## Introduction

The neurotransmitter serotonin (5-HT) exerts a wide spectrum of actions in a variety of behaviors, such as pain sensation, locomotion, circadian rhythm, food intake and emotional behaviors [1], [2]. Extensive efforts have been made to characterize the molecular pathways that control the specification, differentiation and survival of 5-HTergic neurons during brain development [3], because this line of research is very helpful for understanding the genetic basis of central 5-HT deficiency which leads to many mental disorders [4], [5]. Sonic hedgehog secreted from the floor plate triggers the expression of *Mash1* and *GATA2* in progenitor cells in the ventricular zone of hindbrain [6], and both genes are essential for the development of 5-HTergic neurons [7], [8]. 5-HTergic neurons are classified into two groups based on their anatomical location: a rostral group located in the pons and a caudal group located in the medulla oblongata. Although *Nkx2.2* is expressed in the progenitors of all 5-HTergic neurons in hindbrain, evidence from null mutant mice show that it is only required for the generation of 5-HTergic neurons in the dorsal raphe nucleus, one cluster neurons in pons group [9], and *GATA3* is thought to be required for the differentiation of the medulla oblongata group [10]. Both *Lmx1b* and *Pet1* are expressed in postmitotic 5-HTergic neurons and essential for the differentiation and survival of 5-HTergic neurons during embryonic development [4], [11], [12].

Our previous study has shown that *Lmx1b* is persistently expressed in central 5-HTergic neurons during postnatal development and throughout adulthood suggesting that *Lmx1b* may be involved in regulating normal expression of 5-HT in adult brain. To test this hypothesis, we used a tamoxifen-inducible Cre-LoxP system [13] to selectively inactivate *Lmx1b* expression in central 5-HTergic neurons of adult mice. Our data showed that 5-HT level in *Lmx1b* iCKO mice was reduced to 60% of control mice probably due to down-regulation of *Tph2*. In addition, *Sert* and *Vmat2* that are implicated in maintaining normal functions of 5-HTergic neurons were greatly reduced in *Lmx1b* iCKO mice. Thus, *Lmx1b*, an essential gene for the development of central 5-HTergic neurons, is also required for the normal biosynthesis of 5-HT in the adult brain and possibly for regulating normal functions of central 5-HTergic neurons.

## Materials and Methods

### Genetic crossings, genotyping and animal maintenance

*Lmx1b^flox/flox^* mice [14] and *Rosa26-LacZ* reporter (Rosa26R) mice [15] were generated and genotyped as previously described. In *Lmx1b^flox/flox^* mice, exons 4-6 of *Lmx1b* were flanked by two LoxP sites and can be deleted in the presence of Cre *in vivo* [14]. To specifically inactivate *Lmx1b* expression in 5-HTergic neurons in the adult mouse brain, *Lmx1b^flox/flox^* mice were crossed with *Pet1-CreER^T2^ mice* (see below) and their offspring *Pet1-CreER^T2^*; *Lmx1b^flox/+^* mice were then crossed with one another to obtain *Pet1-CreER^T2^*; *Lmx1b^flox/flox^* (*Lmx1b* iCKO) mice. Animal care practices and all experiments were reviewed and approved by the Animal Committee of Tongji University School of Medicine, Shanghai, China (TJmed-010-10).

### Generation of *Pet1-CreER^T2^* mice

Pet1-CreER^T2^ BAC construct was obtained by inserting CreER^T2^ coding sequence downstream of the *Pet1* start codon within the RP23-165D11 BAC (BACPAC Resources Center at Children's Hospital Oakland Research Institute) via homologous recombination in EL250 bacteria [16]. Sepharose-4B (Sigma)-purified BAC DNA was then introduced into FVB/N fertilized mouse eggs by pronuclear injection using standard methods. Transgenic mice were genotyped by PCR with primers against Cre (forward: TCG ATG CAA CGA GTG ATG AG; reverse: TCC ATG AGT GAA CGA ACC TG) resulting in a ∼400 base-pair product. All progeny carrying this transgene were found to be viable and fertile without any obvious abnormalities.

To determine the spatial pattern of Cre activity, *Pet1-CreER^T2^* mice were crossed with *Rosa26R* mice [15] and Cre activity was examined by administering tamoxifen to *Pet1-CreER^T2^*; *Rosa26R* progeny. Tamoxifen (20 mg/ml; Sigma) diluted in corn oil (Sigma) was administered by oral gavage in once-daily doses of 8 mg/40 g of body weight on the following schedule: days 1, 8, 9, 11 and 12. Mice were sacrificed 2-3 weeks after the last dose and brains were removed and fixed in 4% paraformaldehyde (Sigma) in 0.01 M phosphate buffered saline (PBS; pH 7.4) for 3 hours. After cryoprotection with 30% sucrose in PBS, 40 µm-thick sections were cut on a cryostat (CM1900, Leica) and immediately subjected to X-gal staining as described previously [5].

### *In situ* hybridization and immunohistochemistry

*In situ* hybridization probes against *Tph2*, *Sert*, *Aadc* and *Dopamine β-hydroxylase* (*Dbh*), were constructed according to the description on the website of Allen Brain Atlas (http://www.brain-map.org). The *Lmx1b* [17] and *Pet1 in situ* probes encompassed the complete *Lmx1b* and *Pet1* coding sequence, respectively. We also generated an *in situ* probe against exons 4–6 of *Lmx1b* only. All probes were cloned into pGEM-T vector (Promega). Eight mice (4 wild-type and 4 *Lmx1b* iCKO) were used for *in situ* hybridization. For *in situ* hybridization, brains were fixed in 4% PFA in PBS for 24 hours, cryoprotected with 30% sucrose in PBS, and 30 µm-thick transverse sections were cut on a cryostat and mounted onto glass slides (Fisher Scientific). RNA probes labeled by digoxygenin-UTP (Roche) were generated by *in vitro* transcription and hybridization signals were visualized upon nitro blue tetrazolium chloride (Fermentas) and 5-bromo-4-chloro-3-indolyl phosphate (Fermentas) staining.

Twelve mice (6 wild-type and 6 *Lmx1b* iCKO) were used in immunochemistry. Thirty µm-thick brain sections were incubated with primary antibody at 4°C overnight. After washing in PBS, sections were incubated with appropriate secondary antibody for 3 hours at room temperature, washed in PBS, and incubated with Cy3-conjugated streptavidin (1∶1000; Jackson ImmunoResearch) for 1 hour. The following primary antibodies were used: goat anti-β-galactosidase (β-gal; 1∶1000; AbD Serotec), rabbit anti-Lmx1b (1∶2000) [17], rabbit anti-Tph2 (1∶4000) [18], [19], rabbit anti-Vmat2 (1∶1000; Chemicon), mouse anti-Tyrosine hydroxylase (TH; 1∶4000; Sigma). For Tph2 and β-gal double staining, sections were incubated with a mix of the anti-Tph2 and anti-β-gal antibodies overnight, then for 3 hours with a mix of Cy3-labeled donkey anti-rabbit (1∶400; Jackson ImmunoResearch) and biotinylated horse anti-goat IgG (1∶400; Vector Laboratories), and finally with Cy2-conjugated streptavidin (1∶1000; Jackson ImmunoResearch) for 1 hour. There were no immunostaining signals when primary antibodies were omitted or replaced with normal IgG. Stained sections were observed and scanned under a fluorescence or confocal microscope.

### Cell count

We counted positive cells in every six sections. Positive cells in the dorsal raphe nucleus (around the level of −4.72 mm to Bregma) [20] and raphe magnus nucleus (around the level of −4.84 mm to Bregma) were counted for statistical comparison between wild-type and *Lmx1b* iCKO mice (*n* = 4 for each). Statistical significance was determined by the Mann-Whitney test. All data were expressed as mean ± SEM, and error bars represent SEM. *P* values less than 0.05 were considered statistically significant.

### High performance liquid chromatography (HPLC)

Adult (3 months) tamoxifen-induced wild-type and *Lmx1b* iCKO mice were used for HPLC (*n* = 6 for each). Two-three weeks after completion of tamoxifen treatment, whole brains were dissected out immediately after anesthesia with sodium pentobarbital (0.07 mg/g body weight), and HPLC samples were made according to methods described previously [5]. 5-HT and its metabolite 5-hydroxyindoleacetic acid (5-HIAA), dopamine and its metabolite dihydroxyphe-nyacetic acid and homovanillic acid, and norepinephrine were measured using HPLC electrochemical detection as described previously [5]. Statistical significance was determined by the Student's *t*-test. All data were expressed as mean ± SEM, and error bars represent SEM. *P* values less than 0.05 were considered statistically significant.

## Results

### Generation and characterization of *Pet1-CreER^T2^* transgenic mice

**Figure 1:**
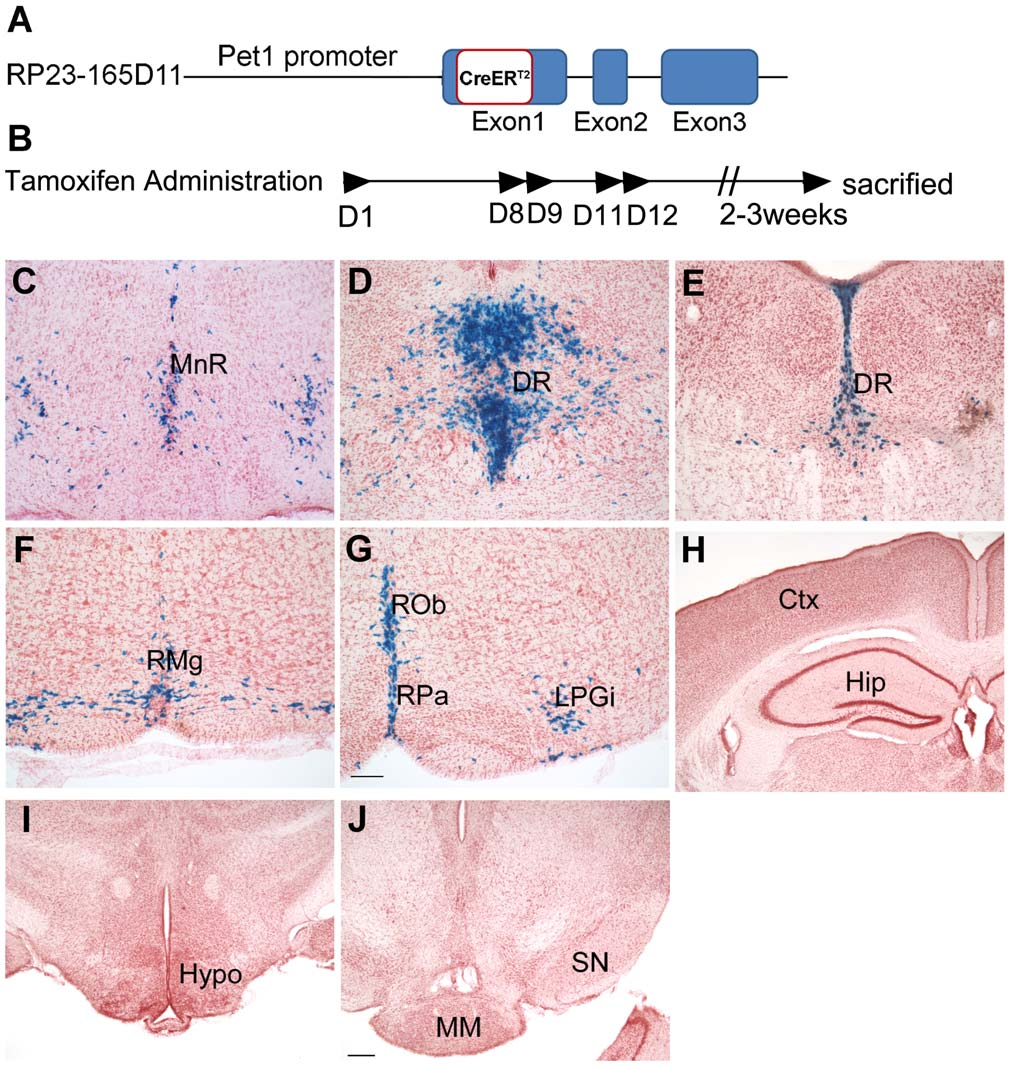
Cre-reombinase activity in *Pet1-CreER^T2^* mice. (A) Diagram of the Pet1-CreER^T2^ construct. The CreER^T2^ coding sequence was inserted in frame into a BAC construct downstream of the *Pet1* start codon by homologous recombination. (B) The procedure for tamoxifen administration. Tamoxifen was administrated on D1, D8, D9, D11 and D12 and mice were sacrificed for analysis 2–3 weeks after the last dose.(C–G). Distribution of X-gal-positive cells in the raphe nuclei of *Pet1-CreER^T2^*; *Rosa26R* mice. (H–J). No X-gal positive cells were found in other brain regions. Ctx, cerebral cortex; DR, dorsal raphe nucleus; Hip, hippocampus; Hypo, hypothalamus; LPGi, lateral paragigantocellular nucleus; MM, medial mammillary nucleus; MnR, median raphe nucleus; RMg, raphe magnus nucleus; ROb, raphe obscurus nucleus; RPa, raphe pallidus nucleus; SN, substantia nigra. Scale bars, 100 µm (B–F) and 200 µm (G–I).

To delete *Lmx1b* in the adult mouse brain, we crossed mice carrying two floxed *Lmx1b* alleles with mice harboring a tamoxifen-inducible form of Cre recombinase (CreERT2) [13] under the control of the Pet1 promoter, to generate *Lmx1b* iCKO mice (Figure 1A). Genotypes were confirmed by PCR. *Pet1* is expressed specifically in central 5-HTergic neurons [4], and the distribution of Cre-recombination activity was determined by crossing to *Rosa26R* mice [15]. *Pet1-CreER^T2^*; *Rosa26R* mice were administered a regimen of tamoxifen (see Materials and methods) beginning at P90 and analyzed by X-gal staining 2–3 weeks after completing the induction regimen. The procedure of tamoxifen administration is shown in Figure 1B. The first day of tamoxifen administration was termed as D1, and tamoxifen was administrated in once-daily doses of 8 mg/40 g body weight on D1, D8, D9, D11 and D12. X-gal-positive cells were found in the raphe nuclei and ventrolateral reticular formation of the medulla oblongata (Figure 1C–G), showing that *Pet1*-driven Cre-recombinase activity can be induced in adulthood. Note that X-gal labeling was not observed in other brain regions outside the raphe nuclei (Figure 1H–J) and no Cre activity was present in negative control-treated and untreated *Pet1-CreER^T2^*; *Rosa26R* mice (data not shown).

**Figure 2:**
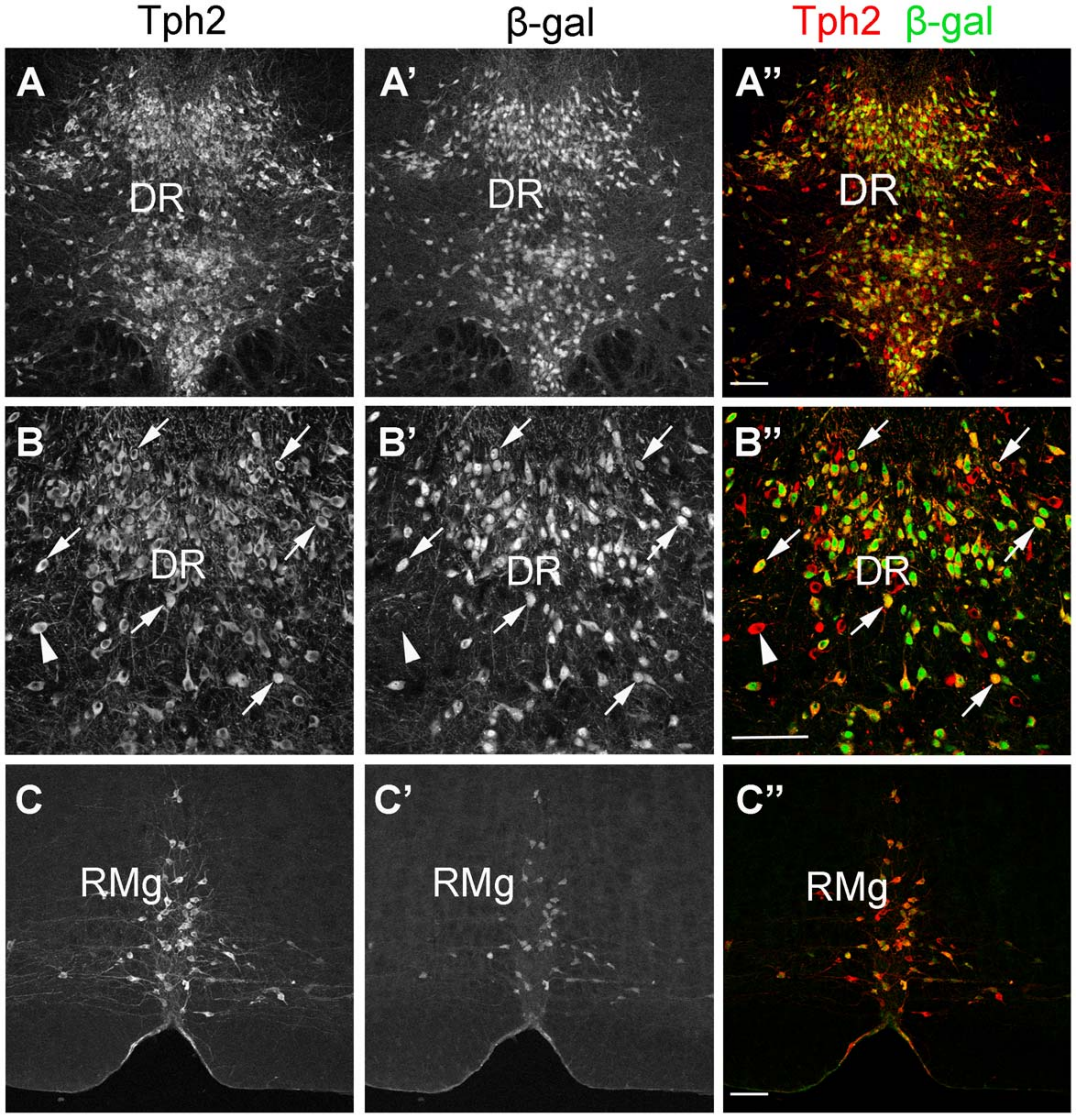
Pet1-CreER^T2^ activity is specific to central 5-HTergic neurons. Double immunolabeling of Tph2 and β-gal in the dorsal raphe nucleus (DR; A-B”) and raphe magnus nucleus (RMg; C-C”) was performed in *Pet1-CreER^T2^; Rosa26R* mice. About 85% of Tph2-positive neurons are co-stained with β-gal antibody (arrows), and a few Tph2-labeled neurons are not β-gal positive (arrowhead). Note that all β-gal-expressing neurons are labeled with Tph2 antibody. B-B” show higher magnifications views of A-A”, respectively. Scale bars, 100 µm.

To determine whether Cre-recombinase activity in *Pet1-CreER^T2^*; *Rosa26R* mice was exclusive to 5-HTergic neurons, we performed Tph2/β-gal double immunostaining. Tph2 is the key enzyme responsible for 5-HT synthesis in the brain [21]. All β-gal-positive neurons expressed Tph2, and approximately 85% of Tph2-labeled neurons were β-gal-positive (Figure 2). Thus, Cre activity in *Pet1-CreER^T2^* mice is specific to 5-HTergic neurons, and is capable of inducing recombination in the majority of 5-HTergic neurons in the adult brain.

### Deleting *Lmx1b* in the adult brain leads to 5-HT insufficiency

Previous studies have demonstrated that *Lmx1b* is required for the differentiation and survival of central 5-HTergic neurons during embryonic development [11], [12]. We set out to examine whether *Lmx1b* also plays a role in the adult central 5-HTergic neurons. *Lmx1b* iCKO and wild-type control mice were administrated with tamoxifen beginning at P90 and examinations were performed 2–3 weeks after completion. We first used Lmx1b antibody [17] to examine whether *Lmx1b* is deleted in *Lmx1b* iCKO mice. As shown in Figure S1, similar Lmx1b immunostaining was found in the dorsal raphe nucleus of wild-type mice and *Lmx1b* iCKO mice showing that the antibody recognizes the truncated Lmx1b protein without exons 4–6 of *Lmx1b*; in this case the Lmx1b antibody can be used to trace *Lmx1b* mutant cells. Because the full length of *in situ* hybridization probe for *Lmx1b* [17] was unable to distinguish truncated mRNA from normal *Lmx1b* mRNA either (data not shown), we generated an *in situ* probe against exons 4–6 of *Lmx1b* only. However, the sensitivity of this probe was too low to detect *Lmx1b* mRNA in adult wild-type mice, although it worked in showing *Lmx1b* mRNA in embryos (data not shown). The floxed *Lmx1b* alleles were deleted after crossing *Lmx1b^flox/flox^* mice with *Pet1*-*Cre* or *Wnt1*-*Cre* mice [5], [14] and Cre activity in *Pet1-CreER^T2^* mice was functional as shown by X-gal staining in 5-HTergic neurons in *Pet1-CreER^T2^*; *Rosa26R* mice (Figures 1, 2), we thus speculate that the exons 4–6 of *Lmx1b* should be deleted in the majority of 5-HTergic neurons in the adult *Lmx1b* iCKO mice (also see phenotypes described below).

**Figure 3:**
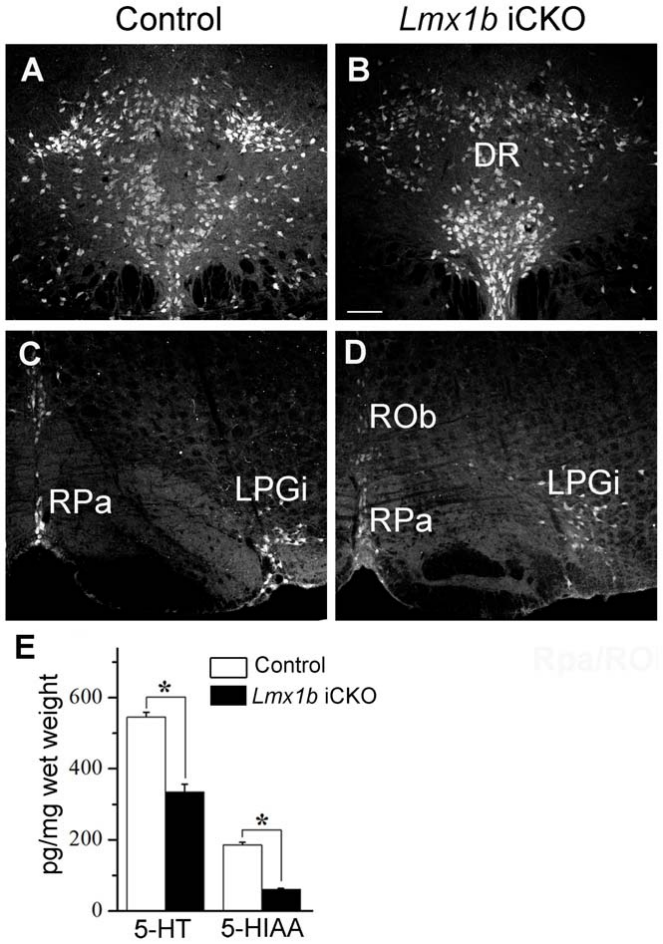
*Lmx1b* is required for the normal expression of 5-HT in the adult brain. (A, B) Intensities of immunofluorescence in most 5-HT-positive neurons in the dorsal raphe nucleus (DR) are reduced in *Lmx1b* iCKO mice (B) relative to that in wild-type controls (A). (C, D) Similar changes in intensities of 5-HT immnofluorescence are also present in the raphe obscurus nucleus (Rob), raphe pallidus nucleus (RPa) and lateral paragigantocellular nucleus (LPGi) of *Lmx1b* iCKO mice relative to those of wild-type mice (C, D). (E) HPLC analysis showing that level of 5-HT and its metabolite 5-HIAA in the brains of *Lmx1b* iCKO mice are reduced to about 60% and 30% of that in controls, respectively (* *P*<0.001). Scale bar, 100 µm.

We next examined 5-HT expression in *Lmx1b* iCKO mice, and found that intensities of 5-HT immunofluorescence in individual neurons were slightly reduced in the raphe nuclei of *Lmx1b* iCKO mice compared with wild-type mice (Figure 3A–D). To further investigate whether the content of 5-HT in the brain is altered or not, we used HPLC to measure the levels of 5-HT and its metabolite 5-HIAA in the brain, and found that they were decreased in *Lmx1b* iCKO mice to about 60% and 30% of control levels, respectively (Figure 3E). We speculate that the discrepancies between no apparent reduction of 5-HT immunofluorescence and 40% reduction of 5-HT revealed by HPLC in *Lmx1b* iCKO are probably due to the low sensitivity of 5-HT antibody, which is unable to detect this reduction. Taken together, we conclude that *Lmx1b* is required for normal expression of 5-HT in adult brain.

### Deleting *Lmx1b* in the adult brain results in down-regulation of 5-HTergic neuron-associated genes

**Figure 4:**
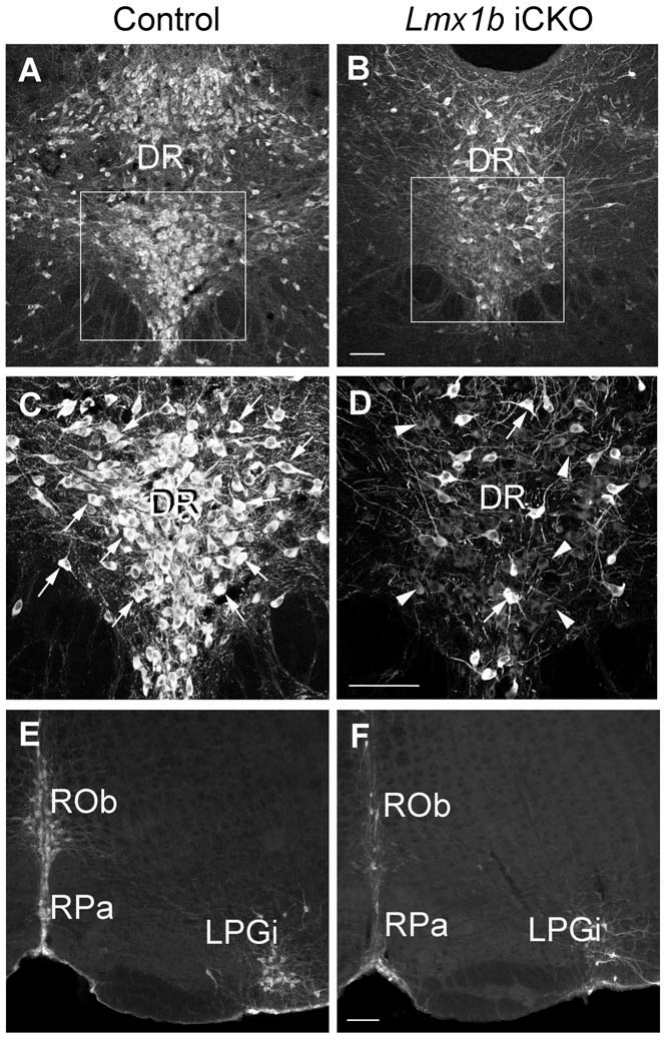
Tph2 expression is reduced in adult *Lmx1b* iCKO mice. (A–D) The number of DR neurons strongly labeled with Tph2 (arrows in C, D) is decreased in *Lmx1b* iCKO mice (B, D) compared with wild-type controls (A, C). C and D are high magnifications of the boxed areas in A and B, respectively. Note that most Tph2-positive neurons in *Lmx1b* iCKO mice are only weakly labeled (arrowheads in D). (E, F) Similar results are observed in the raphe obscurus nucleus (Rob), raphe pallidus nucleus (RPa) and lateral paragigantocellular nucleus (LPGi) of the medulla oblongata. Scale bars, 100 µm.

**Figure 5:**
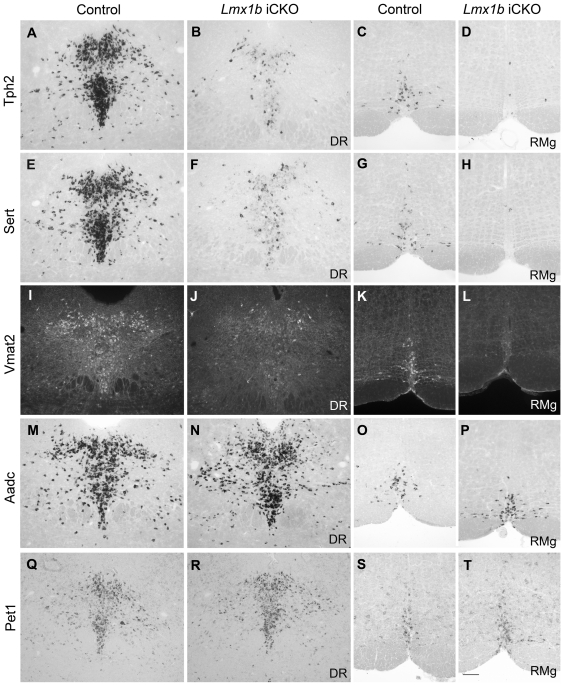
Decreased expression of 5-HT-specific genes in adult *Lmx1b* iCKO mice. (A–L) *Tph2* (A–D) and *Sert* [34] *in situ* hybridization, and Vmat2 (I–L) immunostaining shows down-regulation of these three genes in rostral and caudal raphe nuclei of *Lmx1b* iCKO mice (B, D, F, H, J, L) compared with that in wild-type controls (A, C, E, G, I, K). (M–T) *Aadc* (M–P) and *Pet1* (Q–T) expression is unchanged in *Lmx1b* iCKO raphe nuclei (N, P, R, T) relative to wild-type controls (M, N, Q, S). DR, dorsal raphe nucleus; RMg, raphe magnus nucleus. Scale bar, 100 µm.

**Figure 6:**
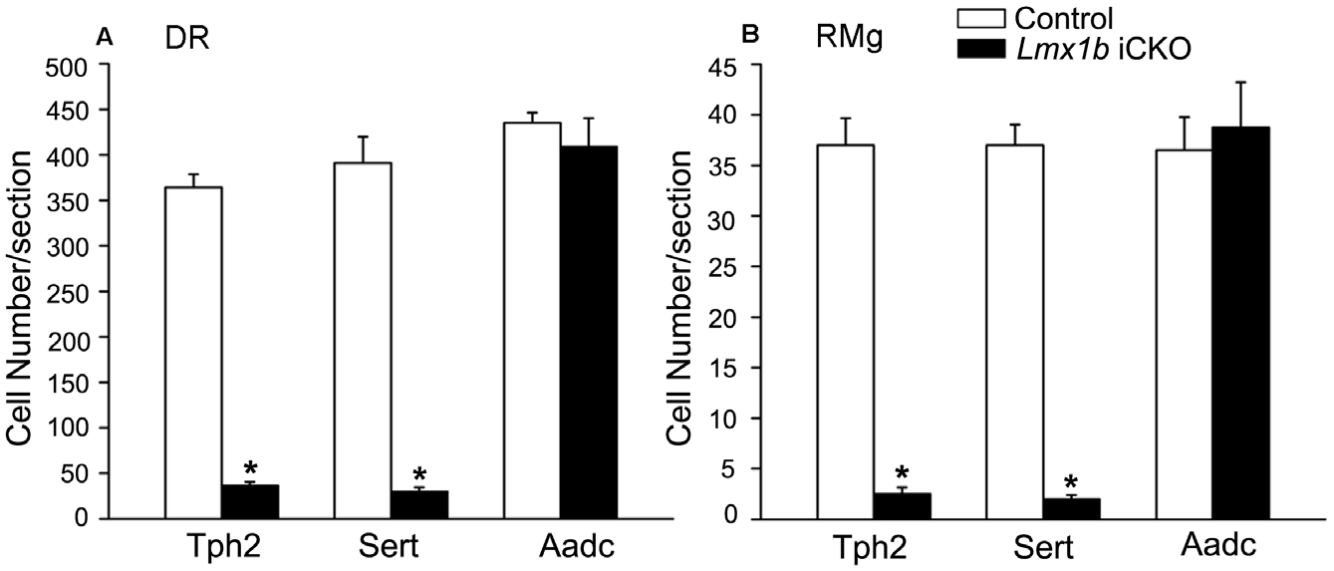
Decrease in numbers of *Tph2*- and *Sert*-labeled neurons in the dorsal raphe nucleus (DR) and raphe magnus nucleus (RMg). The number of neurons with intense *in situ* signals of *Tph2* and *Sert* are significantly decreased in the DR (A) and RMg (B) of *Lmx1b* iCKO mice (n = 4; * *P*<0.05), while the number of *Aadc* positive cell is comparable in *Lmx1b* iCKO mice and wild-type mice (n = 4, *P*>0.5).

To explore the mechanisms underlying decreased 5-HT level in *Lmx1b* iCKO mice, we examined the expression of *Tph2*, which is a specific enzyme for synthesis of 5-HT in the brain [21]. The number of neurons with intense Tph2 immunofluorescence in the raphe nuclei of *Lmx1b* iCKO mice was dramatically reduced compared with control mice (Figure 4). Correspondingly, many weakly-labeled neurons were seen in the *Lmx1b* iCKO raphe nuclei (arrowheads in Figure 4D), whereas they were not observed in wild-type mice. These observations were further confirmed by *in situ* hybridization for *Tph2* (Figure 5A–D). The number of cells with intense *in situ* signals was significantly decreased in *Lmx1b* iCKO relative to wild-type mice (Figure 6). Since approximately 15% of 5-HTergic neurons in *Pet1-CreER^T2^* mice did not exhibit Cre activity (Figure 2), the strong Tph2 labeling retained in *Lmx1b* iCKO mice may correspond to 5-HTergic neurons in which *Lmx1b* was not deleted. Nevertheless, these results indicate that deleting *Lmx1b* in the adult brain impairs *Tph2* expression, leading to a deficiency of central 5-HT.

To further investigate the function of *Lmx1b*, we examined the expression of several genes essential for maintaining the normal function of 5-HTergic neurons. Sert is required for the re-uptake of 5-HT in axonal terminals [22], and its expression was greatly reduced in the raphe nuclei of *Lmx1b* iCKO mice (Figure 5E–H). Cell counts showed a significant difference in the number of *Sert*-expressing cells between wild-type and *Lmx1b* iCKO mice (Figure 6). Vmat2, which is required for packaging 5-HT into synaptic vesicles [23], was also down-regulated in *Lmx1b* iCKO mice (Figure 5I–L). These results indicate that expression of the genes associated with the maintaining functions of 5-HTergic neurons is impaired. To test whether deleting *Lmx1b* decreases the number of 5-HTergic neurons or not, which in turn results in the phenotypes mentioned above, we examined the expression of *Aadc* and *Pet1*. We found that the number of *Aadc*-expressing neurons was unchanged in *Lmx1b* iCKO mice compared with controls (Figures 5M–P, 6), consistent with the finding that similar Lmx1b immunostaining was present in both *Lmx1b* iCKO and wild-type mice (Figure S1). The dorsal raphe nucleus contains the most abundant 5-HTergic neurons among the raphe nuclei. In Nissl-stained sections, the Nissl-stained 5-HTergic neurons are larger and more intensely stained relative to non-5-HTergic neurons. Nissl-stained sections from wild-type and *Lmx1b* iCKO mice showed no obvious difference in cell density and distribution (Figure S2). Furthermore, *Pet1* expression in 5-HTergic neurons requires *Lmx1b* during embryonic development [12], but its expression in the raphe nuclei of *Lmx1b* iCKO mice showed no difference from wild-type controls (Figure 5Q–T). These results suggest that the overall number of 5-HTergic neurons is not affected by deleting *Lmx1b* in adulthood, and that *Pet1* expression in the adult brain is independent of *Lmx1b*.

### Expression of dopamine and norepinephrine is unchanged in *Lmx1b* iCKO mice

**Figure 7:**
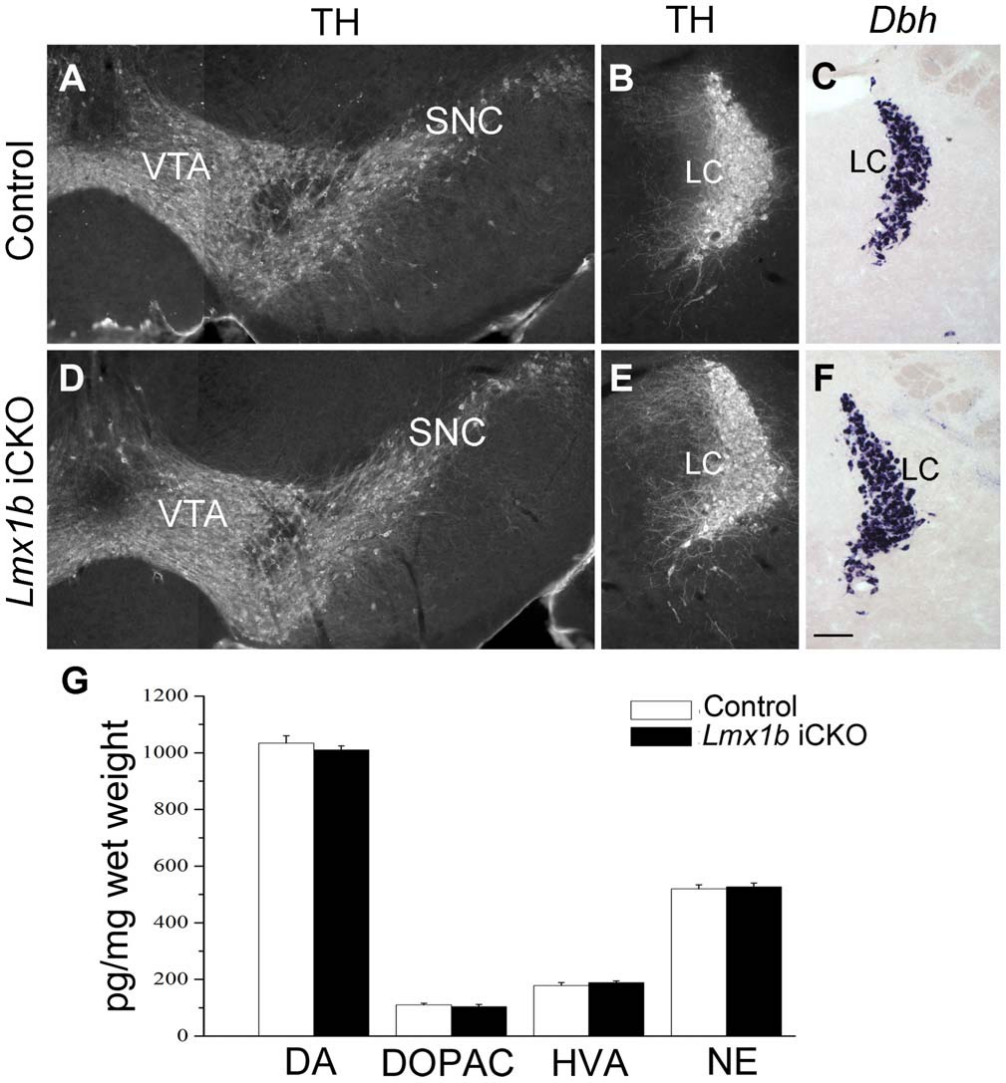
Dopamine (DA) and norepinephrine (NE) expression is unchanged in adult *Lmx1b* iCKO mice. (A, B, D, E) TH immunostaining in the substantia nigra pars compacta (SNC) and ventral tegmental area (VTA) of the midbrain (A, D), and in the locus coeruleus (LC) of the pons (B, E) in *Lmx1b* iCKO mice (D, E) is similar to that of wild-type mice (A, B). (C, F) *In situ* hybridization of *Dbh* in *Lmx1b* iCKO mice (F) is similar to that of wild-type controls (C). (G) Levels of DA, DA metabolites (dihydroxyphenylacetic acid [DOPAC] and homovanillic acid [HVA] and NE in the brains of *Lmx1b* iCKO mice are not different from those of control mice. Scale bar, 100 µm.

revious studies have shown that central 5-HT deficiency may affect the expression of other monoamines in the brain [24]. To explore this possibility, we examined the expression of TH, the essential enzyme for the synthesis of both dopamine and norepinephrine, and *Dbh*, an enzyme that converts dopamine into norepinephrine [25] in *Lmx1b* iCKO mice. TH immunostaining in the substantia nigra and ventral tegmental area (dopaminergic neurons), and in the locus coeruleus (norepinephrinergic neurons) in *Lmx1b* iCKO mice was similar to that in control mice (Figure 7A, B, D, E). *Dbh in situ* hybridization in the locus coeruleus of *Lmx1b* iCKO mice was also similar to that of wild-type controls (Figure 7C, F). In addition, levels of norepinephrine, dopamine and its metabolites (dihydroxyphenylacetic acid and homovanillic acid) in *Lmx1b* iCKO mice were not different from those of controls, as determined by HPLC analysis (Figure 7D). Thus, expression of dopamine and norepinephrine in *Lmx1b* iCKO mice appeared normal.

## Discussion

In the present study, we took advantage of an inducible Cre-LoxP system to inactivate *Lmx1b* expression in adult 5-HTergic neurons. We found that the level of central 5-HT in *Lmx1b* iCKO mice is reduced to 60% of controls, and that the expression of 5-HT neuron-associated genes such as *Tph2*, *Sert* and *Vmat2* are down-regulated in *Lmx1b* iCKO mice.

We generated *Pet1-CreER^T2^* mice, and X-gal staining data from *Pet1-CreER^T2^*; *Rosa26R* mice treated with tamoxifen in adulthood showed that Cre was functional and restricted to central 5-HTergic neurons. Our previous studies have shown that the flanked *Lmx1b* allele was deleted in *Wnt1-Cre; Lmx1b^flox/−^* and *Pet1-Cre; Lmx1b^flox/−^* mice [5], [14]. Although we failed to provide direct morphological data showing the deletion of *Lmx1b*, based on the data mentioned above and phenotypes observed in *Lmx1b* iCKO mice, it is reasonable to speculate that *Lmx1b* is inactivated in 5-HTergic neurons of *Lmx1b* iCKO mice. *Pet1-CreER^T2^* mice are very useful in time-controlled deletion of interested genes in central 5-HTergic neurons particularly in adulthood.

The function of *Lmx1b* in the development of 5-HTergic neurons has been studied extensively [5], [11], [12], [26]. In *Lmx1b* null mice, postmitotic 5-HTergic neurons fail to express 5-HT and several genes (e.g. *Pet1*) critical for 5-HT neuron development [11], [12]. When *Lmx1b* is conditionally deleted after 5-HTergic neuron development has initiated (around embryonic day 12.5), 5-HTergic neurons differentiate normally, but end up dying at later embryonic stages [5], [26]. Thus, *Lmx1b* is required for both differentiation and survival of 5-HTergic neurons during embryonic development. As we showed in the present study, the inactivation of *Lmx1b* in adulthood led to a reduction in central 5-HT levels, probably as a consequence of *Tph2* down-regulation. In addition, Sert, the protein responsible for the re-uptake of 5-HT into axonal terminals, and Vmat2, which is involved in packaging 5-HT into synaptic vesicles [22], [23], were both greatly reduced in the raphe nuclei of *Lmx1b* iCKO mice. In contrast, the expression of both *Pet1* and *Aadc* appeared unchanged in *Lmx1b* iCKO mice relative to control mice, indicating that there was no loss of 5-HTergic neurons in the raphe nuclei. It has been shown that *Pet1* is required for terminal differentiation of 5-HTergic neurons during embryonic development [4], and its expression is lost in *Lmx1b* null mice [12]. Recently, it is reported that *Pet1* is required for maintaining the serotonergic neurotransmitter system during adult stages [27]. Loss of *Pet1* in the 5-HTergic neurons leads to a decrease of Tph2 expression but no change in *Lmx1b* expression. In the present study, normal *Pet1* expression was found in *Lmx1b* iCKO mice. It is likely that *Lmx1b* and *Pet1* act in parallel to regulate central 5-HTergic system, the expression of *Pet1* in adult brain is independent of *Lmx1b*, and *Pet1* is not involved in alterations of gene expression in *Lmx1b* iCKO mice. Taken together, these results indicate that *Lmx1b* is required for 5-HT biosynthesis and expression of several key genes associated with functions of 5-HTergic neurons, but not their survival in adult brain.

Central 5-HT deficiency has been associated with some mental disorders, such as depression and posttraumatic stress disorder [28], [29], [30]. We previously generated *Lmx1b* iCKO mice in which *Lmx1b* is deleted specifically in 5-HTergic neurons at embryonic stage with the help of *Pet1-Cre*, and found that 5-HT level in brain is less than 10% of that in wild-type mice. Interestingly, these mice showed enhanced contextual fear memory [5]. On the other hand, 5-HT plays important roles in the development of nervous system at embryonic stages and during early postnatal development, such as axonal growth [31], spine formation [32] and barrel formation in the somatosensory cortex [33]. It is likely that abrogating 5-HT biosynthesis or 5-HT neuronal development with traditional genetic ablation techniques might have uncontrolled pleiotropic effects by interfering with the development of other brain systems. The use of *Lmx1b* iCKO mice circumvents these complications by allowing the brain to develop normally through to adulthood, and they serve as a new mouse model to study mental disorders associated with central 5-HT deficiency.

## Supporting Information

Figure S1 **The expression of truncated Lmx1b in *Lmx1b* iCKO mice is comparable to that of full‐length Lmx1b in wild-type control.** (A) The expression of full-length Lmx1b in dorsal raphe of wild-type control. (B) The expression of truncated Lmx1b in dorsal raphe of *Lmx1b* iCKO mice. Scale bar, 100 µm.
(DOC)

Figure S2 **Nissl staining shows no difference in the morphological features of the dorsal raphe nucleus between wild‐type and *Lmx1b* iCKO mice.** Scale bar, 100 µm.
(DOC)
