# Supplementary figures and images for: Adult Raphe-Specific Deletion of Lmx1b Leads to Central Serotonin Deficiency
Source: PLoS One. 2011 Jan 5;6(1):e15998. doi: 10.1371/journal.pone.0015998 (PMC3016403; doi:10.1371/journal.pone.0015998)

Supplementary Figure 1


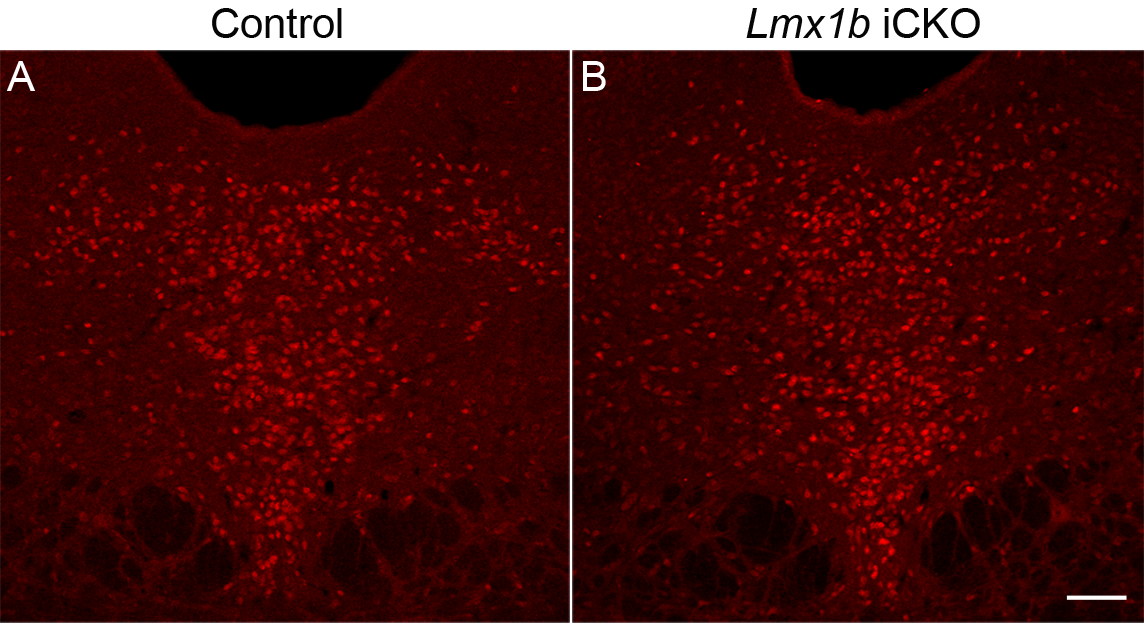

Supplement: Figure S1 — The expression of truncated Lmx1b in Lmx1b iCKO mice is comparable to that of full‐length Lmx1b in wild-type control. (A) The expression of full-length Lmx1b in dorsal raphe of wild-type control. (B) The expression of truncated Lmx1b in dorsal raphe of Lmx1b iCKO mice. Scale bar, 100 µm. (DOC) [file pone.0015998.s001.doc]

Supplementary Figure 2


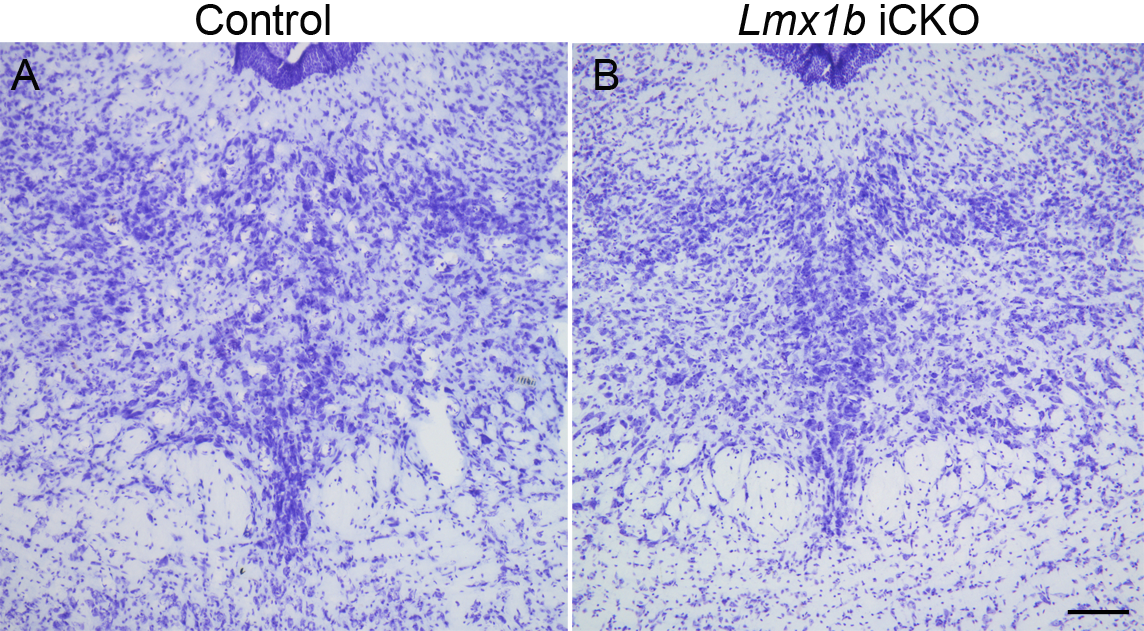

Supplement: Figure S2 — Nissl staining shows no difference in the morphological features of the dorsal raphe nucleus between wild‐type and Lmx1b iCKO mice. Scale bar, 100 µm. (DOC) [file pone.0015998.s002.doc]
